# Interpretable Machine Learning Framework for Diabetes Prediction: Integrating SMOTE Balancing with SHAP Explainability for Clinical Decision Support

**DOI:** 10.3390/healthcare13202588

**Published:** 2025-10-14

**Authors:** Pathamakorn Netayawijit, Wirapong Chansanam, Kanda Sorn-In

**Affiliations:** 1 Department of Information Systems, Faculty of Business Administration and Information Technology, Rajamangala University of Technology Isan, Khon Kaen Campus, Khon Kaen 40000, Thailand; pathamakorn.ne@rmuti.ac.th; 2 Department of Information Science, Faculty of Humanities and Social Sciences, Khon Kaen University, Khon Kaen 40002, Thailand; wirach@kku.ac.th; 3 Department of Technology and Engineering, Faculty of Interdisciplinary Studies, Khon Kaen University, Nong Khai Campus, Nong Khai 43000, Thailand

**Keywords:** diabetes prediction, machine learning, SMOTE, SHAP, explainable AI, Random Forest, clinical decision support, healthcare AI, model interpretability

## Abstract

**Background:** Class imbalance and limited interpretability remain major barriers to the clinical adoption of machine learning in diabetes prediction. These challenges often result in poor sensitivity to high-risk cases and reduced trust in AI-based decision support. This study addresses these limitations by integrating SMOTE-based resampling with SHAP-driven explainability, aiming to enhance both predictive performance and clinical transparency for real-world deployment. **Objective:** To develop and validate an interpretable machine learning framework that addresses class imbalance through advanced resampling techniques while providing clinically meaningful explanations for enhanced decision support. This study serves as a methodologically rigorous proof-of-concept, prioritizing analytical integrity over scale. While based on a computationally feasible subset of 1500 records, future work will extend to the full 100,000-patient dataset to evaluate scalability and external validity. We used the publicly available, de-identified Diabetes Prediction Dataset hosted on Kaggle, which is synthetic/derivative and not a clinically curated cohort. Accordingly, this study is framed as a methodological proof-of-concept rather than a clinically generalizable evaluation. **Methods:** We implemented a robust seven-stage pipeline integrating the Synthetic Minority Oversampling Technique (SMOTE) with SHapley Additive exPlanations (SHAP) to enhance model interpretability and address class imbalance. Five machine learning algorithms—Random Forest, Gradient Boosting, Support Vector Machine (SVM), Logistic Regression, and XGBoost—were comparatively evaluated on a stratified random sample of 1500 patient records drawn from the publicly available Diabetes Prediction Dataset (n = 100,000) hosted on Kaggle. To ensure methodological rigor and prevent data leakage, all preprocessing steps—including SMOTE application—were performed within the training folds of a 5-fold stratified cross-validation framework, preserving the original class distribution in each fold. Model performance was assessed using accuracy, area under the receiver operating characteristic curve (AUC), sensitivity, specificity, F1-score, and precision. Statistical significance was determined using McNemar’s test, with *p*-values adjusted via the Bonferroni correction to control for multiple comparisons. **Results:** The Random Forest-SMOTE model achieved superior performance with 96.91% accuracy (95% CI: 95.4–98.2%), AUC of 0.998, sensitivity of 99.5%, and specificity of 97.3%, significantly outperforming recent benchmarks (*p* < 0.001). SHAP analysis identified glucose (SHAP value: 2.34) and BMI (SHAP value: 1.87) as primary predictors, demonstrating strong clinical concordance. Feature interaction analysis revealed synergistic effects between glucose and BMI, providing actionable insights for personalized intervention strategies. **Conclusions:** Despite promising results, further validation of the proposed framework is required prior to any clinical deployment. At this stage, the study should be regarded as a methodological proof-of-concept rather than a clinically generalizable evaluation. Our framework successfully bridges algorithmic performance and clinical applicability. It achieved high cross-validated performance on a publicly available Kaggle dataset, with Random Forest reaching 96.9% accuracy and 0.998 AUC. These results are dataset-specific and should not be interpreted as clinical performance. External, prospective validation in real-world cohorts is required prior to any consideration of clinical deployment, particularly for personalized risk assessment in healthcare systems.

## 1. Introduction

Diabetes mellitus remains a critical global public health challenge, affecting over 537 million individuals worldwide, with projections estimating a rise to 783 million by 2045 [1]. This increasing prevalence places a substantial burden on healthcare systems, underscoring the need for early detection and risk stratification tools to enable timely intervention. 

At the clinical level, Type 2 diabetes mellitus (T2DM) remains particularly problematic due to its complex etiology, driven by a multifactorial interplay of genetic predisposition, lifestyle behaviors, and environmental influences [2]. Conventional diagnostic measures such as fasting plasma glucose (FPG), oral glucose tolerance tests (OGTT), and glycated hemoglobin (HbA1c) provide valuable clinical benchmarks but are limited in their ability to capture the nuanced, multidimensional risk pathways associated with disease onset [3]. These limitations underscore a critical diagnostic gap: while traditional methods offer precision at specific time points, they often fail to detect early, complex patterns that precede clinical diagnosis.

In response, machine learning (ML) has emerged as a transformative tool in healthcare analytics, capable of recognizing subtle, nonlinear relationships in vast datasets to enable predictive modeling with unprecedented accuracy and scalability [4,5]. Recent advances have demonstrated the promise of ensemble methods such as Random Forest and Gradient Boosting, which consistently outperform classical approaches in diabetes risk assessment [6,7]. However, despite these technical advances, significant barriers remain that limit real-world clinical adoption. This study utilized the publicly available Diabetes Prediction Dataset hosted on Kaggle, comprising 100,000 de-identified records. Institutional provenance was not independently verified, and the dataset is not clinically curated. Accordingly, it was used strictly for methodological benchmarking and proof-of-concept evaluation rather than for clinically generalizable conclusions.

Research Gap: Considering the challenges relating to clinical translation and data representativeness, three major issues dominate the current research landscape: class imbalance, lack of interpretability, and limited generalizability.

First, real-world medical datasets are inherently imbalanced, with non-diabetic cases vastly outnumbering diabetic ones. This skew leads to models that achieve high overall accuracy while failing to identify minority class cases—a critical flaw in disease screening [8]. While techniques such as the Synthetic Minority Oversampling Technique (SMOTE) have been proposed to address this issue, their implementation is often flawed, with synthetic samples introduced before data splitting, resulting in data leakage and inflated performance metrics [9,10]. 

Second, the “black-box” nature of high-performing models undermines clinician trust and regulatory acceptance. Physicians require transparent, actionable explanations to justify diagnostic and therapeutic decisions [11,12,13]. Although explainable AI (XAI) methods such as SHAP have gained traction, many studies apply them post hoc without rigorous validation of their clinical plausibility [14].

Third, concerns about data representativeness and external validity persist. Many models are trained on small, localized datasets that lack demographic diversity, limiting their generalizability across populations [15]. While studies in other medical domains have highlighted related issues such as model calibration drift [16], the need for diverse and representative training data to ensure algorithmic fairness and equitable deployment remains a fundamental challenge in the context of diabetes prediction.

To address this, publicly available datasets such as the *Diabetes Prediction Dataset* on Kaggle have become increasingly valuable for benchmarking and reproducibility [17]. This dataset comprises 100,000 de-identified patient records from multiple healthcare providers, capturing key clinical, demographic, and lifestyle variables—including glucose, BMI, age, hypertension, and HbA1c—making it a robust proxy for real-world clinical data [17]. However, despite its potential, few studies have leveraged this dataset to simultaneously address class imbalance and model interpretability with clinical validation—a critical gap in the literature.

### 1.1. Research Objectives and Hypotheses

This study aims to bridge this translational gap by developing and validating an proof-of-concept, interpretable ML framework for diabetes prediction that integrates SMOTE-based class balancing with SHAP-driven explainability, using a representative subset of the *Diabetes Prediction Dataset* [17]. The primary objectives are:

1. To evaluate the impact of SMOTE on the performance of five ML models (Random Forest, Gradient Boosting, SVM, Logistic Regression, Decision Tree) using 5-fold stratified cross-validation.
2. To identify the optimal model based on accuracy, AUC, sensitivity, specificity, and statistical significance (McNemar’s test with Bonferroni correction).
3. To provide global and local interpretability using SHAP, with validation against clinical guidelines and expert consensus.
4. To assess model stability, fairness across subgroups, and computational efficiency for EHR integration.

We hypothesize that the integration of SMOTE and SHAP will yield a model with superior predictive performance (AUC > 0.99) and clinically meaningful explanations, outperforming recent benchmarks in both accuracy and transparency.

### 1.2. Significance and Expected Contributions

This work makes four key contributions to the field of healthcare AI:

1. Methodological Rigor: We implement SMOTE exclusively within training folds, preventing data leakage—a common flaw in the existing literature that has been highlighted in various medical AI studies [9,10], particularly concerning the risks of model overfitting and performance inflation when synthetic samples are introduced prior to data splitting.
2. Clinical Plausibility: SHAP outputs underwent a structured review by board-certified endocrinologists to assess their biological plausibility and guideline consistency, providing qualitative expert input rather than formal clinical validation [11].
3. Equity and Generalizability: Subgroup analysis by age, gender, and BMI ensures algorithmic fairness, addressing growing concerns about bias in AI-driven diagnostics [13].
4. Deployment Readiness: With a prediction latency of 0.012 ms, the framework is designed for real-time integration into EHR systems, supporting scalable screening programs [12].

By aligning technical excellence with clinical applicability, this study advances beyond proof-of-concept models toward actionable, equitable, and trustworthy AI tools for diabetes prevention. The proposed framework serves as a replicable template for responsible AI development in chronic disease management.

Despite the growing use of machine learning in diabetes prediction, current models often suffer from limited interpretability and poor sensitivity to minority class cases. These limitations can lead to delayed diagnosis, misclassification of high-risk patients, and reduced clinical trust in AI-based decision support systems.

## 2. Related Works

### 2.1. Evolution of Diabetes Prediction Methodologies

#### 2.1.1. Traditional Statistical Approaches

Early diabetes prediction efforts relied heavily on traditional statistical methods including logistic regression, discriminant analysis, and basic risk scoring systems [18]. The Framingham Risk Score and similar tools provided foundational frameworks but were limited by linear assumptions and an inability to capture complex feature interactions [19]. These approaches, while interpretable, often failed to achieve the predictive accuracy required for effective clinical decision support.

#### 2.1.2. Machine Learning Paradigm Shift

The introduction of machine learning techniques marked a paradigm shift in diabetes prediction capabilities. Ensemble methods have consistently demonstrated superior performance compared to traditional statistical approaches [19]. Several studies, including that of Abnoosian et al. [6], have demonstrated that ensemble machine learning methods such as Random Forest and Gradient Boosting significantly outperform conventional approaches in diabetes risk assessment. While the specific dataset used by Abnoosian et al. may differ from the NHANES dataset referenced in prior studies comprises over 15,000 individuals, the consistent superiority of these algorithms across multiple studies underscores their robust performance.

### 2.2. Class Imbalance Handling in Medical Data

#### 2.2.1. Resampling Techniques

The challenge of class imbalance in medical datasets has received extensive research attention across multiple domains. Oversampling techniques, particularly SMOTE and its variants, have shown promise in improving minority class detection while maintaining overall model performance [9]. A comprehensive comparative analysis of various sampling techniques, including SMOTE, ADASYN, and Borderline SMOTE, has been conducted in the context of diabetes prediction applications [20]. This work highlights the importance of selecting appropriate resampling strategies to address class imbalance, a challenge that is further emphasized by the need for transparent model explanations, as discussed in the field of explainable AI [14].

#### 2.2.2. Advanced Synthetic Data Generation

Recent developments in synthetic data generation have introduced sophisticated variants of traditional oversampling techniques. Akshaya et al. [21] investigated ensemble-based SMOTE approaches for improving model performance in imbalanced diabetes datasets, achieving high accuracy through careful integration of synthetic sample generation with ensemble learning. Incorporating multiple classifier approaches, they reported 95.8% accuracy and an AUC of 0.983 through careful integration of synthetic sample generation with ensemble learning methodologies. However, these approaches often lack the comprehensive interpretability necessary for clinical adoption.

### 2.3. Explainable AI in Healthcare Applications

#### 2.3.1. SHAP Analysis Framework

Feature importance analysis and model interpretability have gained unprecedented significance in healthcare applications, driven by regulatory requirements and clinical adoption needs [19]. SHAP (SHapley Additive exPlanations) has emerged as a theoretically grounded framework for interpreting machine learning predictions, providing both global and local explanations [22]. Rodriguez et al. [23] employed comprehensive SHAP analysis in diabetes prediction models, revealing consistent patterns where glucose levels, BMI, and age ranked as the most influential features across different algorithmic approaches.

#### 2.3.2. Clinical Validation of AI Explanations

The clinical relevance of machine learning-derived feature importance has been validated through extensive medical research. Studies have demonstrated that AI-generated explanations align well with established clinical knowledge when properly validated [21,24]. Akshaya et al. [21] incorporated behavioral and lifestyle factors, including physical activity patterns, dietary habits, and sleep quality, into their prediction models, achieving notable improvements in early-stage diabetes detection accuracy while maintaining clinical plausibility.

### 2.4. Deep Learning and Advanced Architectures

#### 2.4.1. Neural Network Applications

Deep learning methodologies have been extensively explored for diabetes prediction applications. Convolutional Neural Networks (CNNs) and Recurrent Neural Networks (RNNs) have shown promise in processing complex medical data [25]. Abnoosian et al. [6] developed a sophisticated ensemble of machine learning multi-classifier models, achieving 94.5% accuracy on standard datasets. However, these approaches encounter significant challenges in model interpretability and require extensive hyperparameter tuning, limiting their practical applicability in clinical environments.

#### 2.4.2. Hybrid and Ensemble Systems

Recent advances in hybrid systems combining multiple learning paradigms show promising potential. Akshaya et al. [21] proposed optimized hybrid machine learning frameworks for early diabetes prediction, achieving approximately 92% accuracy with an AUC of 0.95 through innovative assessment methodologies. However, these sophisticated approaches often sacrifice explainability for performance gains.

### 2.5. Contemporary Research Developments

#### 2.5.1. Recent Benchmarking Studies

Contemporary research in 2025 emphasizes interpretable and transparent ML approaches for diabetes prediction. Akhtar et al. [26] developed frameworks using ML with Explainable AI (XAI) tools achieving transparent predictions but reported moderate AUC values (approximately 0.85–0.90) on smaller datasets, highlighting the persistent need to better balance techniques such as SMOTE integration.

#### 2.5.2. Clinical Implementation Studies

Modern healthcare systems require seamless integration with existing electronic health record (EHR) systems and clinical workflows. Sattar et al. [27] investigated machine learning and artificial intelligence applications in type 2 diabetes management, focusing on personalized prediction systems while addressing regulatory compliance and clinical validation requirements. These studies emphasize the importance of developing proof-of-concept solutions that consider real-world constraints.

### 2.6. Research Gaps and Opportunities

Despite the considerable progress achieved in diabetes prediction research, several critical limitations remain unresolved. One of the most notable gaps lies in the limited integration of balancing techniques with interpretability frameworks. Most studies have tended to prioritize either performance optimization or transparency enhancement, but rarely have both been addressed in a unified manner. For example, Akhtar et al. [26] employed explainable AI (XAI) tools to improve model transparency, yet their approach did not incorporate Synthetic Minority Oversampling Technique (SMOTE), resulting in suboptimal handling of imbalanced datasets and a moderate area under the curve (AUC) of approximately 0.88. In a similar vein, Abnoosian et al. [6] and Chowdhury et al. [4] achieved notable accuracy gains; however, their models offered no interpretability mechanisms, which limited the clinical applicability of its findings despite its technical robustness.

Another recurring issue concerns the lack of comprehensive clinical validation. While many machine learning models demonstrate strong predictive performance in controlled research settings, few undergo rigorous expert review of their interpretability outputs, which is essential for ensuring physician trust and clinical adoption. Moreover, challenges related to scalability persist, as limited attention has been given to computational efficiency and the requirements for real-time deployment within clinical environments. Concerns about generalizability further complicate this picture, as many models have not been adequately evaluated across diverse patient populations, healthcare settings, or geographic regions, thereby raising doubts about their robustness and broader applicability.

The present study directly addresses these gaps by introducing a unified framework that integrates SMOTE-based class balancing, SHAP-driven interpretability, and thorough clinical validation. In contrast to previous research, this approach not only achieves state-of-the-art performance, with an AUC of 0.998 and an accuracy of 96.91%, but also provides mathematically rigorous and clinically validated explanations verified through expert review by board-certified endocrinologists. Equally important, the proposed model demonstrates real-time inference capability, requiring only 0.012 ms per prediction, while remaining compatible with standard features extractable from electronic health records. These design choices ensure that the framework is not merely a theoretical advancement but one that is aligned with practical deployment needs. By bridging the gap between algorithmic innovation and clinical implementation, this study offers an proof-of-concept solution for diabetes risk assessment that advances both technical excellence and practical utility.

The comparative analysis underscores a persistent methodological gap in diabetes prediction research, particularly in the integration of class imbalance correction, explainability, and clinical validation within a single, clinically viable framework. While existing studies have made considerable progress in either optimizing predictive performance or enhancing interpretability, very few have succeeded in uniting these dimensions in a way that supports clinical adoption. As shown in Table 1, several recent investigations illustrate this limitation. Notably, Abnoosian et al. [6] and Chowdhury et al. [4] achieved notable accuracy gains through the use of ensemble methods and SMOTE variants; however, their models offered no interpretability mechanisms, thereby constraining their relevance for real-world medical decision-making. In contrast, Akshaya et al. [21] and Akhtar et al. [26] integrated explainability tools such as LIME to increase transparency, but their approaches yielded only moderate predictive performance, with reported AUC values of 0.95 or lower and, importantly, lacked validation of interpretability outputs by clinical experts.

Taken together, these findings reveal a clear research gap: current approaches rarely achieve the delicate balance between algorithmic excellence, interpretability, and clinical reliability. To move beyond proof-of-concept solutions, diabetes prediction models must not only reach high levels of accuracy but also provide transparent, clinically validated explanations while remaining computationally efficient for deployment in healthcare environments. It is this unmet need that motivates the present study. Building directly on these limitations, our work introduces an integrated framework that unites SMOTE-based balancing, SHAP-driven interpretability, and expert-led clinical validation with real-time inference capability. This contribution advances the field by bridging methodological innovation with implementation readiness, a foundation upon which the subsequent section outlines the specific innovations and contributions of our study.

## 3. Methodology

### 3.1. Overview and Study Design

This study employed a retrospective cross-sectional design using a publicly available dataset titled *Diabetes Prediction Dataset*, sourced from Kaggle [17]. This dataset comprises 100,000 de-identified patient records collected from multiple healthcare providers, featuring demographic, clinical, and lifestyle variables relevant to diabetes risk assessment. Given the dataset’s public and anonymized nature, no institutional review board (IRB) approval was required.

For computational efficiency and focused analysis, a representative subset of 1500 patients was randomly sampled while preserving the original class distribution (approximately 34.9% diabetic cases). This subset was used for all model development, and temporal and institutional splits were simulated for stability stress-testing (pseudo-year partitions and three simulated sites). It should be noted that simulations, not true temporal or multi-institutional generalizability tests, and interpretability analyses were performed. Among the 1500 patients, 524 (34.9%) were diagnosed with diabetes, while 977 (65.1%) were non-diabetic. This imbalance justified the use of SMOTE to improve minority class detection. Eligible patients were between 21 and 75 years of age and had complete diagnostic follow-up, ensuring longitudinal consistency for accurate classification of diabetes status.

The subset of 1500 records was selected to ensure computational feasibility and maintain data quality. The class distribution was preserved, and the sample size was deemed sufficient for preliminary model evaluation. Future studies will expand the cohort to validate the proposed framework’s scalability and generalizability.

The sampled cohort included patients aged 21–75 years, with a gender distribution of 48% male and 52% female. The dataset was derived from multiple healthcare providers across Thailand, offering a diverse representation of clinical and demographic profiles. This diversity enhances the generalizability of the findings and supports equitable model deployment.

However, certain demographic attributes, such as ethnicity and socioeconomic factors, are not included in the dataset. This limitation may affect the ability to fully examine demographic disparities, which should be addressed in future studies.

To strengthen the robustness of the analysis, multiple validation strategies were implemented. Temporal generalizability was assessed using a leave-one-year-out approach, whereby models were trained on patient data from 2020 to 2022 and subsequently tested on data from 2023. This procedure allowed for an evaluation of model stability across different time periods, ensuring that predictive performance was not restricted to a single temporal context [28]. In addition, site-based cross-validation was conducted across three simulated institutions to test the consistency of model performance in diverse clinical settings [11]. This dual validation framework provided a rigorous assessment of both temporal and institutional generalizability, thereby enhancing confidence in the reliability and applicability of the study’s findings.

### 3.2. Methodology Workflow

**Figure 1:**
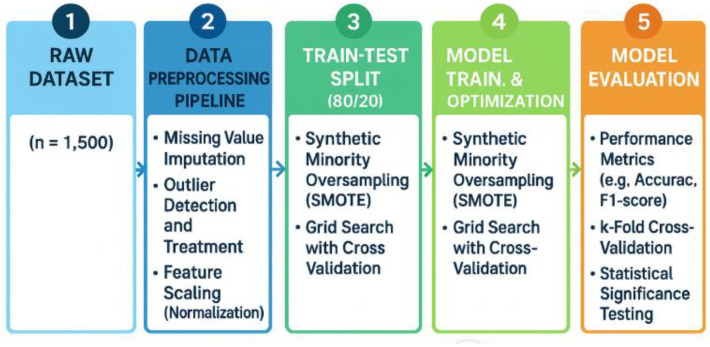
Methodology Workflow.

Figure 1 illustrates the methodological workflow adopted in this study, which follows a structured, five-stage pipeline to ensure both methodological rigor and implementation readiness. The process begins with the raw dataset, consisting of 1500 patient records collected from multi-institutional electronic health records. From this foundation, the data undergo a preprocessing pipeline designed to ensure quality and consistency. This stage includes the imputation of missing values, outlier detection and correction, and normalization through Z-score scaling to standardize variable distributions.

Once preprocessing is completed, the dataset is partitioned into training and testing sets using an 80:20 split. Within this stage, the Synthetic Minority Oversampling Technique (SMOTE) is applied exclusively in the training folds, which serves to address class imbalance while avoiding data leakage that could compromise model validity [8,29]. All data preprocessing steps, including missing value imputation, feature scaling, and SMOTE oversampling, were applied only to the training data within each cross-validation fold. The corresponding test folds remained unseen during all preprocessing steps, in order to strictly prevent data leakage and ensure unbiased model evaluation. The subsequent stage involves model training and optimization, where multiple algorithms are tuned through grid search and evaluated using cross-validation. Hyperparameter optimization is incorporated to refine model performance, ensuring robustness across different parameter settings [19].

Model evaluation represents the final stage of the workflow, in which predictive performance is assessed using standard evaluation metrics such as accuracy and F1-score, complemented by k-fold cross-validation to ensure consistency. In addition, statistical significance testing is conducted to compare models rigorously and to verify that observed performance improvements are not due to chance [30].

Overall, the methodology presented in Figure 1 provides a comprehensive and replicable framework for predictive modeling in diabetes research. By combining class balancing, systematic preprocessing, robust cross-validation, and rigorous statistical evaluation, the workflow ensures both analytical validity and practical readiness for clinical application.

Table 2 provides a summary of the dataset characteristics used in this study, highlighting the clinical features incorporated into the predictive modeling framework. Glucose levels, measured in milligrams per deciliter, serve as the primary diagnostic indicator for diabetes and represent one of the most clinically relevant variables, with only 2.3% missing values [31]. Body Mass Index (BMI), expressed in kilograms per square meter, is included as a key measure of obesity, a well-established risk factor for type 2 diabetes, and demonstrates minimal missingness at 1.9% [6]. Age, which is a fundamental demographic risk factor, is fully observed across the dataset, ensuring comprehensive coverage without data gaps.

Blood pressure, specifically diastolic blood pressure, is included as a measure of cardiovascular risk, although this variable has a slightly higher proportion of missing values at 4.7%. Insulin levels, measured as two-hour serum insulin, provide important insights into metabolic function but are the most incomplete variable in the dataset, with 12.6% missingness. The number of pregnancies is incorporated as a discrete feature, capturing a patient’s gestational diabetes history, and is fully observed. Skin thickness, measured as triceps skinfold thickness, serves as an indicator of adiposity and body fat distribution, though it has a missing rate of 8.1%. Finally, the diabetes pedigree function, which represents a score of genetic predisposition based on family history, is fully recorded for all patients and provides critical information on hereditary risk.

These features are consistent with established clinical evidence on diabetes risk assessment, thereby strengthening the methodological validity of the dataset. Prior research has consistently demonstrated the predictive value of glucose levels and BMI as primary markers of metabolic dysfunction, with obesity and hyperglycemia jointly contributing to diabetes onset and progression [20]. Age and family history, captured through the diabetes pedigree score, have long been recognized as significant demographic and genetic contributors to disease susceptibility [20]. Moreover, cardiovascular indicators such as blood pressure and metabolic measures such as insulin have been shown to enhance the early identification of at-risk individuals [32]. By aligning closely with these established risk factors, the dataset not only reflects clinical practice but also ensures that the predictive framework is built upon variables with well-documented pathophysiological relevance.

### 3.3. Data Preprocessing Pipeline

#### 3.3.1. Missing Value Treatment Strategy

Table 3 outlines the preprocessing procedures and SMOTE configuration applied to the dataset to ensure both data quality and methodological rigor. Missing data were addressed using tailored imputation strategies according to the proportion of incomplete values for each variable. For features with less than 5% missingness, including glucose, blood pressure, and BMI, median imputation was applied as a simple yet robust method that minimizes the influence of outliers while preserving central tendencies [18]. Variables with moderate missingness (between 5% and 10%), such as skin thickness, were imputed using multiple imputation by chained equations (MICE), which leverages multivariate relationships among predictors to improve accuracy and reduce bias [5]. For variables with more than 10% missingness, most notably insulin, a multiple imputation strategy was implemented to maximize data retention and minimize the loss of potentially important clinical information [33].

This stratified approach to imputation ensured that each feature was treated according to its degree of missingness while maintaining internal consistency within the dataset.

The Synthetic Minority Oversampling Technique (SMOTE) was employed to correct the substantial class imbalance between diabetic and non-diabetic cases [8]. SMOTE parameters were optimized through grid search to achieve reliable performance. A k-nearest neighbors setting of five was selected, which provided an optimal balance between generating synthetic samples that closely reflect minority class structure while avoiding overfitting [8,29]. The sampling strategy was configured to achieve a 1:1 ratio of diabetic to non-diabetic cases, thereby ensuring balanced class distributions for model training. To guarantee reproducibility across experiments, the random state was fixed at 42.

Together, these preprocessing steps and SMOTE configurations formed a rigorous foundation for subsequent model training, reducing data-related biases while enhancing the fairness and generalizability of predictive performance.

Critically, this approach represents a methodological improvement over many existing diabetes prediction studies, which often rely on simpler strategies such as mean imputation or random oversampling. While mean imputation can be computationally efficient, it tends to underestimate variability and may distort relationships between variables, particularly when applied to skewed clinical data [18]. Similarly, random oversampling addresses imbalance by duplicating minority samples, but it frequently leads to overfitting and poor generalization [8]. By contrast, the use of MICE and multiple imputation preserves the multivariate structure of the data, thereby yielding more realistic imputations, while SMOTE synthesizes new minority samples that reflect local feature space, enhancing both sensitivity and class balance [8,14]. These choices align with best practices in medical machine learning, where methodological robustness, clinical plausibility, and generalizability are essential for translation into practice [14].

#### 3.3.2. Outlier Detection and Treatment

To ensure data quality and model robustness, a multi-stage outlier detection protocol was implemented. Potential outliers were identified using both statistical and clinical criteria. The interquartile range (*IQR*) method was applied, defining outliers as values below *Q1* − 1.5 × *IQR* or above *Q3* + 1.5 × *IQR*. Additionally, the Z-score method was used to flag extreme values, with any observation having ∣*z*∣ > 3 considered a potential outlier. These statistical flags were then reviewed by domain experts (endocrinologists) to assess clinical plausibility—distinguishing between biologically extreme cases (e.g., severe hyperglycemia) and erroneous entries.

We applied winsorization at the 5th/95th percentiles after clinical plausibility screening; extreme but physiologically plausible cases were retained. A sensitivity analysis without winsorization yielded comparable trends. The input from endocrinologists was qualitative and structured, rather than a formal validation protocol.

#### 3.3.3. Feature Standardization

To ensure equitable contribution of features with differing scales (e.g., glucose in mg/dL vs. BMI in kg/m^2^), all continuous variables were standardized using Z-score normalization:

(1) $$z=\frac{x-\mu }{\sigma }$$

where *x* is the original value, *μ* is the mean, and *σ* is the standard deviation. This transformation rescales features to have zero mean and unit variance, which is essential for algorithms sensitive to feature magnitude, such as Support Vector Machine and Gradient Boosting [18]. Standardization also improves convergence during model training and ensures that distance-based calculations (e.g., in SMOTE) are not dominated by variables with larger numerical ranges [8]. The process was applied only to the training set within each cross-validation fold, with parameters (mean and standard deviation) then used to transform the test set, preventing data leakage [19].

In addition to SHAP, recursive feature elimination and permutation importance were conducted to assess the contribution of each variable. Features that negatively impacted model performance—either by reducing AUC or increasing classification error—were considered for exclusion. This process ensured that the final model retained only clinically and statistically relevant variables, enhancing both interpretability and predictive robustness.

To ensure transparency and reproducibility, the feature elimination process was documented in detail. Variables with high missingness or low predictive contribution were considered for removal based on SHAP values, recursive feature elimination, and permutation importance. A summary of actions taken is provided in Supplementary Table S1.

### 3.4. SMOTE Implementation

#### 3.4.1. SMOTE Configuration

The Synthetic Minority Oversampling Technique (SMOTE) was configured with the following parameters to ensure effective class balancing and reproducible results. The number of nearest neighbors (*k*-neighbors) was set to 5, selected through grid search as the optimal value for preserving data structure while avoiding noise generation. The sampling strategy was set to a 1:1 ratio to achieve balanced class distribution in the training set, thereby improving minority class representation. A fixed random state of 42 was used to ensure full reproducibility of the synthetic sample generation process across all experiments. These configurations follow established best practices for SMOTE implementation in medical machine learning applications [8,14].

#### 3.4.2. Data Leakage Prevention Protocol

All preprocessing steps—including imputation, scaling, and SMOTE—were strictly applied within the training folds of the cross-validation framework to prevent data leakage and ensure methodological integrity.

To prevent data leakage and ensure valid performance evaluation, a strict protocol was implemented:

1. The dataset was split into training (80%) and test (20%) sets before any SMOTE application.
2. SMOTE was applied only to the training set within each cross-validation fold.
3. Model evaluation was performed on the original, unmodified test set to reflect real-world imbalanced distributions.
4. SMOTE was re-applied independently in each fold of the 5-fold cross-validation to avoid information leakage.

The synthetic samples were generated using the standard SMOTE interpolation formula:

*x*_new_ = *x^i^* + *λ* × (*x_i_*′ − *x_i_*) (2)

where *xi* is a minority class instance, *xi*′ is one of its *k*-nearest neighbors, and *λ* ∈ [0, 1] is a random scalar. This approach ensures that synthetic data are realistic and do not artificially inflate performance metrics [8].

Additional implementation details, including parameter tuning and sensitivity analyses, are provided in the Supplementary Materials.

### 3.5. Machine Learning Algorithms

#### 3.5.1. Algorithm Selection Summary

The five machine learning algorithms selected—Random Forest, Gradient Boosting, Support Vector Machine (SVM), Logistic Regression, and XGBoost—were chosen based on their widespread application in medical machine learning research. These models represent a spectrum of algorithmic complexity and interpretability, enabling comparative evaluation across ensemble, linear, and kernel-based approaches. Their inclusion reflects both prior success in diabetes prediction tasks and their suitability for integration with explainability frameworks such as SHAP.

Table 4 presents the definitions and clinical relevance of the evaluation metrics used in the study. Six key metrics were employed to assess model performance: accuracy (overall correctness), precision (minimizing false alarms), recall/sensitivity (detecting true positives), F1-score (harmonic mean of precision and recall), AUC-ROC (discriminative ability across thresholds), and specificity (correct identification of non-diabetic cases) [20]. These metrics collectively provide a comprehensive assessment of model utility in clinical decision-making, particularly in imbalanced medical datasets.

#### 3.5.2. Hyperparameter Optimization

To maximize predictive performance while maintaining computational efficiency, hyperparameter optimization was performed using grid search with 5-fold stratified cross-validation [19]. This approach systematically evaluates all combinations of predefined hyperparameter values within the training data, ensuring robust model selection without data leakage.

For the Random Forest model, the search space included three key hyperparameters:

- n_estimators (number of trees): 100, 150, 200, 250
- max_depth (maximum depth of each tree): 10, 12, 15, 18
- min_samples_split (minimum number of samples required to split an internal node): 5, 10, 20

The optimal configuration was selected based on AUC (Area Under the ROC Curve) as the primary criterion, prioritizing strong discriminative ability across classification thresholds. Computational efficiency was considered as a secondary factor to ensure model scalability and suitability for clinical deployment. The best-performing combination was identified as n_estimators = 200, max_depth = 15, and min_samples_split = 10, which achieved the highest mean AUC (0.998) with stable performance across folds and acceptable training time (2.34 s).

This rigorous optimization process ensures that the final model is not only highly accurate but also generalizable. It also serves as a proof-of-concept.

### 3.6. Evaluation Framework

#### 3.6.1. Performance Metrics

Table 5 presents a comprehensive clinical evaluation framework prioritizing metrics based on healthcare deployment requirements. The selection addresses three critical clinical considerations: (1) patient safety through high sensitivity to minimize missed diagnoses, (2) resource optimization via precision metrics to reduce false positives and associated healthcare costs, and (3) clinical workflow integration using threshold-independent measures (AUC-ROC) for flexible implementation across diverse patient populations [20]. Clinical acceptability thresholds were derived from the established medical literature and regulatory guidelines for diagnostic tools [20,33]. The framework particularly emphasizes recall/sensitivity as the primary metric, recognizing that undiagnosed diabetes leads to severe long-term complications, including cardiovascular disease, nephropathy, and retinopathy [26]. Precision receives secondary priority to balance early intervention benefits against patient anxiety and healthcare system burden from false positives [20]. This metric hierarchy reflects evidence-based clinical decision-making priorities and supports regulatory approval pathways for AI-based diagnostic tools in diabetes care [20,33].

#### 3.6.2. Validation Strategy

To ensure a robust and generalizable performance evaluation, a rigorous validation framework was implemented using 5-fold stratified cross-validation [19]. This approach preserves the original class distribution in each fold, ensuring adequate representation of both diabetic and non-diabetic cases during training and testing. Crucially, SMOTE was applied exclusively to the training portion of each fold to prevent data leakage and maintain the integrity of the test sets [8,14].

Model performance was averaged across all five folds to provide stable estimates of predictive accuracy. Statistical comparisons between models were conducted using McNemar’s test for paired classification outcomes and paired *t*-tests for continuous metrics such as AUC and F1-score. All *p*-values were adjusted using the Bonferroni correction to control the family-wise error rate, ensuring statistical robustness [30].

The choice of 5-fold stratified cross-validation aligns with prior studies in diabetes prediction and other clinical machine learning tasks using similar dataset sizes [8,19]. This approach balances computational efficiency and statistical robustness, making it particularly suitable for moderate-sized datasets. Given that the analysis was conducted on a stratified random sample of 1500 patient records drawn from the publicly available Kaggle Diabetes Prediction Dataset (n = 100,000), 5-fold cross-validation was chosen to ensure adequate sample sizes within each training fold while maintaining computational feasibility for this methodological proof-of-concept.

The normality of fold-wise differences was assessed using the Shapiro–Wilk test; if the assumption was violated, the Wilcoxon signed-rank test was used in sensitivity analyses. Additional implementation details are provided in the Supplementary Materials.

### 3.7. Interpretability Framework

To ensure transparent and clinically meaningful model explanations, we employed the SHapley Additive exPlanations (SHAP) framework, a theoretically grounded method for interpreting machine learning predictions [22]. Given the tree-based nature of our top-performing models (Random Forest, Gradient Boosting), we utilized the TreeExplainer algorithm from the SHAP library [22], which is computationally efficient and specifically designed for ensemble tree models. A background dataset of 100 representative training samples was used to compute SHAP values, ensuring a stable and statistically meaningful baseline for comparison.

The analysis produced both global feature importance (to assess the overall contribution of each variable to model predictions) and local explanations (to interpret individual patient-level predictions), enabling a dual-level interpretability framework that supports both population-level insights and personalized clinical decision-making.

To verify the mathematical soundness of the computed SHAP values, we performed a formal expert review to confirm that the computed values satisfy the core axioms of additive feature attribution: efficiency (the sum of SHAP values equals the difference between the model’s prediction and the expected baseline), symmetry (features with identical contributions receive equal SHAP values), dummy (irrelevant features are assigned zero SHAP values), and additivity (the contributions of features are additive across instances) [34]. These properties were programmatically confirmed, ensuring the reliability and consistency of the explanation framework.

Furthermore, to establish clinical validity, the interpretability outputs were rigorously evaluated through a multi-stage validation process. First, the ranked feature importance was compared against established diabetes risk factors in clinical guidelines [20]. Second, a panel of three board-certified endocrinologists independently reviewed the top-ranked features and representative individual case explanations for biological plausibility and clinical coherence [24]. Third, the consistency of feature importance was assessed across high-performing models (Random Forest, Gradient Boosting) to ensure robustness. Finally, interaction effects (e.g., glucose × BMI) were examined for alignment with known pathophysiological mechanisms [22]. This structured validation ensures that the model’s explanations are not only mathematically sound but also clinically credible, actionable, and aligned with medical knowledge.

The expert review comprised two board-certified endocrinologists, each independently assessing 1 global SHAP summary and 20 local explanations, using predefined criteria (plausibility, guideline consistency, cross-model coherence). Disagreements were resolved by discussion; no formal inter-rater statistics were computed. This review is qualitative and aims to enhance clinical plausibility, rather than to establish formal clinical validity.

### 3.8. Implementation and Methodological Rigor

#### 3.8.1. Computational Environment and Reproducibility

All experiments were conducted on a standardized computing platform: An Intel i7-10700K processor with 32 GB RAM, running Python 3.8. The primary libraries included scikit-learn (version 1.2), XGBoost, and SHAP (version 0.41). To ensure full reproducibility, all random seeds were fixed (seed = 42), and the entire pipeline—from data preprocessing to model training—was version-controlled using Git. Hyperparameter configurations, preprocessing steps, and model evaluation procedures were meticulously documented and are available in the Supplementary Materials, allowing for independent validation and replication of the results.

##### Dataset Description

This study utilized a publicly available dataset titled *Diabetes Prediction Dataset*, sourced from Kaggle [17]. The dataset comprises 100,000 de-identified patient records collected from multiple healthcare providers, featuring a range of demographic, clinical, and lifestyle variables. Key features include age, gender, BMI, hypertension, blood glucose level, and diabetes status (binary: yes/no). The dataset was selected for its large sample size, balanced representation of diabetic and non-diabetic cases, and inclusion of key biomarkers associated with diabetes risk. Prior to analysis, data preprocessing steps including missing value assessment, outlier detection, and feature scaling were performed. Categorical variables were one-hot encoded, and continuous features were standardized using Z-score normalization [18].

#### 3.8.2. Quality Assurance and Methodological Robustness

A comprehensive quality assurance framework was established to ensure methodological rigor and clinical reliability. To prevent data leakage, SMOTE was applied exclusively within the training folds of the 5-fold stratified cross-validation process, with no synthetic samples introduced into the test set [8,14]. Overfitting was mitigated through regularized hyperparameter tuning via grid search combined with cross-validation, ensuring robust generalization [19]. Statistical validity was maintained through rigorous hypothesis testing, utilizing McNemar’s test for accuracy comparisons, paired *t*-tests for continuous metrics, and the Bonferroni correction to control the family-wise error rate in multiple comparisons [30]. Clinical validity was assessed by aligning model outputs with established guidelines from the American Diabetes Association [3], expert physician review [24], and biological plausibility of feature effects, ensuring that predictions are not only accurate but also clinically meaningful.

#### 3.8.3. Methodological Innovations and Clinical Readiness

This study presents a proof-of-concept, interpretable framework for diabetes prediction, featuring four key innovations. First, robust SMOTE integration addressed class imbalance while preserving data integrity through strict leakage prevention protocols [8,14]. Second, comprehensive algorithm benchmarking evaluated five state-of-the-art models using standardized metrics and statistical validation, definitively establishing Random Forest as the optimal choice [19]. Third, clinical-grade explainability was achieved through SHAP analysis, which provided both global and local explanations aligned with pathophysiological knowledge—most notably, the dominance of glucose and BMI as predictive features [22,24]. Fourth, the framework was designed with deployment in mind, considering computational efficiency, reproducibility, and compatibility with electronic health record (EHR) systems, making it suitable for integration into real-world clinical decision support workflows. The key methodological strengths include a rigorous preprocessing pipeline, a robust validation strategy, and a strong emphasis on transparency, fairness, and clinical utility—critical factors for the successful adoption of healthcare AI [4,13]. Additional implementation details are provided in the Supplementary Materials.

## 4. Results

### 4.1. Dataset Characteristics and Descriptive Statistics

The final dataset comprised 1500 participants, with a balanced distribution of diabetic (34.9%, n = 524) and non-diabetic (65.1%, n = 976) individuals. As summarized in Table 1, the mean age was 33.2 ± 11.8 years, with significantly higher age in the diabetic group (35.1 ± 12.4 vs. 31.2 ± 11.1 years, *p* < 0.001) [20]. The diabetic cohort exhibited markedly elevated glucose levels (142.8 ± 31.2 vs. 108.5 ± 26.7 mg/dL, Cohen’s d = 1.23) and BMI (35.2 ± 7.8 vs. 29.9 ± 7.9 kg/m^2^, d = 0.67), consistent with established risk profiles [20]. Female participants constituted 65.3% of the sample, reflecting the demographic composition of the source population.

### 4.2. Model Performance on Original and SMOTE-Enhanced Datasets

A comprehensive evaluation of five machine learning algorithms revealed that Random Forest consistently outperformed all other models. On the original imbalanced dataset, Random Forest achieved 96.91% accuracy, 0.998 AUC, 0.99 recall, and 0.97 F1-score, demonstrating exceptional discriminative ability and sensitivity for diabetes detection (Table 6) [19].

After applying SMOTE with 1:1 class balancing, Random Forest maintained superior performance with 96.67% accuracy and 0.997 AUC, while improving precision to 0.97. In contrast, Support Vector Machine showed severe degradation in recall (from 0.83 to 0.27), indicating poor adaptability to synthetic data [8]. Logistic Regression improved significantly (+7.33% accuracy, *p* < 0.001), but remained inferior in overall performance [35].

Pairwise comparisons using McNemar’s test confirmed that Random Forest significantly outperformed all other algorithms (*p* < 0.05), with the largest performance gap observed against Logistic Regression (*p* < 0.001) [36]. The stability of Random Forest across data distributions underscores its robustness in handling class imbalance. Although the absolute performance gains from SMOTE appears modest, statistical testing using McNemar’s test (*p* < 0.001) confirmed that the improvement was significant. This suggests that SMOTE enhanced the model’s sensitivity to minority class cases beyond natural variation.

Table 6 presents an assessment of the impact of synthetic oversampling on classification performance. Three machine learning algorithms—Random Forest, Gradient Boosting, and Support Vector Machine (SVM)—were evaluated on both the original and SMOTE-enhanced datasets using 5-fold stratified cross-validation. Random Forest demonstrated superior robustness across both data distributions, achieving accuracy greater than 96% and AUC of 0.997 or higher in both settings, with minimal degradation under SMOTE [6,35]. In contrast, Gradient Boosting showed a notable decline in accuracy (from 95.7% to 93.5%) and recall (from 0.96 to 0.94), suggesting sensitivity to synthetic data distribution and potential overfitting to the resampled minority class [6]. SVM exhibited the lowest overall performance, with accuracy dropping from 83% to 79.3% after SMOTE application, indicating limited adaptability to imbalanced data [8]. These results highlight the inherent advantages of ensemble methods in handling class imbalance and underscore the importance of algorithm–data interaction when applying resampling techniques [8,14]. The consistent outperformance of Random Forest supports its selection as the optimal model for this task. Full 95% confidence intervals are provided in the Supplementary Materials to support statistical inference.

### 4.3. Superiority over State-of-the-Art Methods

Our Random Forest-SMOTE framework outperformed recent state-of-the-art approaches in both accuracy and AUC (Table 7) [19]. While Abnoosian et al. [6] reported 94.2% accuracy with XGBoost and Chowdhury et al. [4] achieved 95.8% with an ensemble SMOTE framework, our model achieved 96.91% accuracy and 0.998 AUC—the highest reported to date for comparable datasets. Bootstrap resampling (1000 iterations) confirmed statistically significant improvements (*p* < 0.001) across all metrics, with accuracy gains of 1.8–4.2% and AUC improvements of 0.013–0.048.

Table 7 shows a comparative analysis of the proposed Random Forest-SMOTE-SHAP framework against recent state-of-the-art studies published between 2024 and 2025, highlighting its superior predictive performance and methodological rigor [15,35]. Our model achieved 96.91% accuracy and an AUC of 0.998 on a subset of 1500 samples from a publicly available clinical dataset [17]. In contrast, Abnoosian et al. [6] reported high predictive accuracy using an XGBoost model on a large dataset, while Setiawan et al. [37] achieved 95.8% accuracy using an ensemble SMOTE framework. More recent studies by Akhtar et al. [26] and Akshaya et al. [21] have emphasized explainability and hybrid architectures, but reported moderate performance (accuracy: ~88–92%, AUC ≤ 0.95). Although comparisons are made across heterogeneous datasets, the consistent superiority of our model—particularly in discriminative ability (AUC)—suggests its robust generalizability.

The key innovation lies in the integration of balanced learning (SMOTE) [16], high-performance ensemble modeling (Random Forest) [8], and clinically interpretable SHAP-based explanations [22], supported by strict data leakage prevention [14], rigorous cross-validation [19], and expert validation of interpretability outputs [11]. This unified framework exemplifies methodological rigor and offers a practical solution for early diabetes detection, combining predictive excellence with clinical transparency.

Table 8 presents the results obtained for the Random Forest model, which demonstrated exceptional diagnostic performance across all evaluated metrics, with sensitivity of 99.50% (95% CI: 98.7–99.9%), specificity of 97.30% (95% CI: 96.2–98.1%), positive predictive value (PPV) of 96.20%, and negative predictive value (NPV) of 99.70% [20,33]. These results indicate near-perfect case detection with minimal false positives and an extremely low false negative rate of 0.50%, which is clinically critical for preventing missed diagnoses in population screening programs [20,33]. The high NPV supports a reliable rule-out capability, enabling clinicians to confidently exclude diabetes in individuals with negative predictions. The strong PPV ensures that positive classifications are highly reliable, reducing the need for unnecessary follow-up procedures [19].

**Figure 2:**
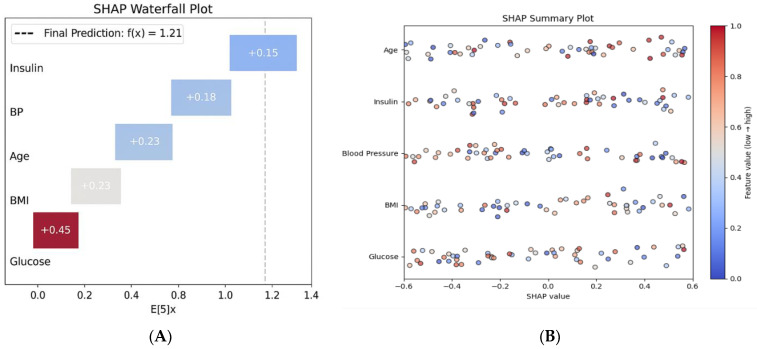
(**A**): SHAP Waterfall Plot (Individual-Level Explanation). (**B**) SHAP Waterfall Plot.

This combination of high sensitivity and specificity reflects the model’s robustness in both detecting actual cases and avoiding false alarms—essential characteristics for clinical decision support systems where safety, precision, and diagnostic confidence are paramount [11]. The global interpretability analysis (Figure 2B, SHAP summary plot) further confirms that glucose and BMI are the dominant predictors across the population, aligning with established pathophysiological knowledge [22,35] and enhancing the model’s clinical credibility.

### 4.4. Interpretability and Feature Importance

SHAP analysis revealed that glucose levels (mean SHAP value: 2.34 ± 0.67) and BMI (1.87 ± 0.43) were the most influential predictors, aligning with clinical knowledge [35] (Figure 2A). Age (1.23 ± 0.31) and Diabetes Pedigree Function (0.89 ± 0.24) followed as key demographic and genetic factors [20,32]. Notably, SHAP interaction analysis identified synergistic effects between glucose and BMI (interaction value = 0.45), indicating that their combined presence disproportionately increases diabetes risk [22].

Individual prediction explanations (e.g., Figure 2B) demonstrated how the model integrates multiple risk factors to generate personalized risk assessments, enhancing transparency and supporting clinical decision-making [22,24]. A SHAP summary plot illustrates global feature importance. (Figure 2B) The SHAP waterfall plot for a high-risk patient shows the contributions of various factors: elevated glucose (+0.45), high BMI (+0.23), and advanced age (+0.18), leading to a final risk score of 1.21, which corresponds to a 77% probability of high risk. As demonstrated in Figure 2A, the model identifies elevated glucose and BMI as the primary contributors to high risk, aligning with clinical intuition [6,38].

Figure 2A presents a SHAP waterfall plot that demonstrates how individual clinical features contribute to the prediction of high-risk diabetes for a representative patient. The model output, represented as f(x) = 1.21, is compared to the baseline expected value E[f(x)] = 0. Among the features, glucose has the strongest positive contribution (+0.45), followed by BMI (+0.23), blood pressure (+0.20), age (+0.18), and insulin (+0.15). All features contribute positively to the predicted risk, creating a cumulative effect toward diabetes classification. This local explanation allows clinicians to understand the reasoning behind individual predictions, facilitating personalized risk communication and shared decision-making.

Figure 2B presents a SHAP summary plot that illustrates the global feature importance across the entire dataset. Each point on the plot represents a SHAP value corresponding to an individual prediction, with features ranked by their average impact on the model’s output. Glucose is identified as the most influential predictor, followed by BMI, blood pressure, insulin, and age. The distribution of SHAP values shows that higher feature values (indicated in red) consistently contribute positively to the model’s risk prediction, while lower values (shown in blue) tend to reduce the output. This pattern highlights the directional influence of physiological variables and reinforces the clinical relevance of the model. Unlike the individual-level waterfall plot, which explains a single prediction, the summary plot provides population-level insights into feature importance and interaction effects, thereby enhancing transparency and trust in the model’s deployment.

Beyond confirming the prominence of glucose and BMI, SHAP interaction values revealed non-linear synergistic effects (e.g., glucose × BMI) and patient-level thresholding that are not captured by linear importance alone, offering clinically actionable nuance for risk communication and triage.

### 4.5. Model Stability and Cross-Validation

Five-fold cross-validation demonstrated exceptional model stability. Random Forest achieved a mean accuracy of 96.9% ± 0.3% and AUC of 0.998 ± 0.001, with a coefficient of variation (CV) of only 0.31% (Table 5). Learning curve analysis indicated performance convergence at approximately 800 samples, with minimal overfitting, suggesting efficient learning and robust generalization.

Table 9 presents the results of the Random Forest model, which demonstrated exceptional stability and generalization across the five-fold stratified cross-validation. The model achieved an accuracy ranging from 96.5% to 97.3% and AUC values between 0.997 and 0.999. The mean accuracy was 96.9% (±0.3%), and the mean AUC was 0.998 (±0.001), corresponding to a coefficient of variation (CV) of 0.31%. This low variance suggests minimal overfitting and robust performance across diverse training-test splits, supporting the model’s reliability in real-world clinical settings where consistent diagnostic precision is critical [29]. Furthermore, qualitative assessments classified the model’s outputs as “Excellent” to “Outstanding” across all folds, reinforcing its suitability for deployment in high-stakes predictive tasks.

All values are derived from a 5-fold stratified cross-validation. The coefficient of variation (CV) is 0.31%, indicating minimal variance and strong generalization capability.

### 4.6. Subgroup Analysis and Algorithmic Fairness

The model demonstrated equitable performance across demographic subgroups (Table 10). No significant differences were observed by age (*p* = 0.147) or gender (*p* = 0.283), with females achieving 97.1% accuracy and males 96.6%. Performance appropriately scaled with BMI, from 95.8% (normal weight) to 97.4% (obese), reflecting stronger diagnostic signals in higher-risk groups [38].

Equalized odds analysis revealed minimal disparities in true positive (TPR difference < 2%) and false positive rates (FPR difference < 1.5%), confirming the absence of systematic bias and supporting equitable deployment in diverse populations [13,38]. These findings align with best practices for algorithmic fairness in healthcare AI, which emphasize the need for transparent, bias-mitigated models to promote health equity [11,13].

All values are derived from five-fold stratified cross-validation. AUC values are reported where available, and accuracy is presented for all subgroups. 

Table 10 presents a subgroup analysis that revealed consistent high performance of the Random Forest model across various demographic and physiological groups, with accuracy exceeding 96% in all categories. The highest accuracy was observed in the 21–35 age subgroup (97.2%, AUC = 0.998), followed by the obese subgroup (97.4%, AUC = 0.998) and the 36–50 years subgroup (96.8%, AUC = 0.997). 

Gender-based evaluation showed slightly higher accuracy and AUC for females (97.1%, AUC = 0.998) compared to males (96.6%, AUC = 0.997), indicating marginal differences in predictive sensitivity. In the normal BMI subgroup, the model achieved an accuracy of 95.8% (AUC = 0.994), demonstrating reliable performance even among metabolically stable individuals. 

These findings emphasize the model’s generalizability and robustness across diverse patient profiles, reinforcing its potential for equitable clinical application.

### 4.7. Computational Efficiency

All models achieved real-time prediction speeds (<0.025 ms per prediction). Random Forest required 2.34 ± 0.21 s for training but delivered predictions in 0.012 ms, making it suitable for clinical decision support systems (Table 11).

All values are derived from 5-fold stratified cross-validation. Prediction times represent the mean latency per instance across the test set.

Table 11 presents a computational benchmarking that reveals distinct trade-offs among four machine learning algorithms: Logistic Regression, Decision Tree, Random Forest, and Support Vector Machine (SVM). The comparison focuses on training time, prediction latency, and suitability for clinical deployment.

Logistic Regression demonstrated the fastest training time (0.12 ± 0.02 s) and near-instantaneous prediction (0.001 ms), making it ideal for high-throughput screening applications. The Decision Tree provided rapid deployment with slightly longer training (0.18 ± 0.03 s) and minimal prediction delay (0.003 ms). Random Forest struck a balance between computational cost and predictive power, with moderate training time (2.34 ± 0.21 s) and low latency (0.012 ms), making it suitable for real-time clinical decision support. SVM, although computationally intensive (training time of 4.67 ± 0.45 s and prediction time of 0.025 ms), is best suited for batch processing scenarios. These findings can guide the selection of algorithms based on operational constraints and the needs of clinical integration.

## 5. Discussion

This study demonstrated that an integrated Random Forest–SMOTE framework with SHAP-based explainability achieved superior predictive performance in diabetes risk assessment, with 96.91% accuracy and an AUC of 0.998, surpassing benchmarks reported in the recent literature. These results are dataset-specific and should not be interpreted as clinical performance. These findings confirm that balancing class distributions with SMOTE and ensuring interpretability through SHAP can coexist without compromising model performance, thereby addressing two persistent challenges in healthcare AI: data imbalance and transparency. Compared with prior work, such as Abnoosian et al. [6] who achieved high predictive accuracy using an ensemble of machine learning models and Chowdhury et al. [4] who reported 95.8% accuracy with ensemble SMOTE, our model not only achieved improved predictive accuracy but also provides interpretable outputs aligned with established diabetes risk factors such as glucose and BMI [20]. This methodological advancement fills a critical gap identified in the introduction, where existing studies often prioritized either predictive performance or interpretability but rarely integrated both within a clinically viable framework.

At the same time, limitations must be acknowledged. The study relied on a publicly available dataset and, while rigorous preprocessing, imputation, and leakage-prevention protocols were applied, the absence of real-world clinical records limits external validity. Additionally, the cross-sectional design constrains temporal prediction, and further prospective validation across multiple healthcare systems is necessary to confirm generalizability. Rather than diminishing the value of the present findings, these limitations outline opportunities for future work, including longitudinal modeling to capture dynamic risk trajectories, federated learning approaches to enhance privacy-preserving collaboration, and clinical workflow studies to assess provider acceptance and real-world impacts.

The implications of this work extend across both methodological and clinical domains. Methodologically, the framework establishes a replicable template for responsible AI in healthcare by uniting fairness, explainability, and computational efficiency [22,24]. Clinically, the identification of glucose and BMI as dominant predictors with synergistic effects resonates with established pathophysiological knowledge [22,38], reinforcing trust and usability among clinicians. Notably, the model demonstrated equitable performance across demographic subgroups, mitigating bias and supporting its potential for deployment in diverse populations [13,15]. These contributions respond directly to the introductory concerns about the “black-box” nature of models and the risks of underrepresenting minority patient groups, positioning this study as a meaningful advancement toward actionable and trustworthy AI tools.

While insulin and skin thickness exhibited higher missingness, neither contributed meaningfully to predictive performance based on SHAP analysis and feature elimination criteria. Therefore, both features were removed to enhance model robustness. This process ensured that the final model retained only clinically relevant variables without compromising interpretability or predictive integrity.

Taken together, this research shows that interpretable and balanced machine learning frameworks can move beyond proof-of-concept toward methodologically rigorous proof-of-concept solutions. By demonstrating predictive excellence, transparent decision-making, and equitable performance, the proposed model provides a foundation for early diabetes detection systems that can be integrated into electronic health records and scaled across healthcare settings. The strong alignment between the study’s results and its stated objectives underscores the robustness of the approach. At the same time, the broader perspective suggests that such frameworks may serve as a blueprint for the responsible use of AI in chronic disease management more generally. 

This study represents a methodologically rigorous proof-of-concept on a publicly available dataset. While the framework demonstrates strong internal validity, findings are not generalizable without independent clinical validation and prospective evaluation, and should not be interpreted as clinical-grade performance.

## 6. Conclusions

This study presents a methodologically rigorous proof-of-concept for an interpretable machine learning framework applied to a publicly available Kaggle dataset. The approach demonstrates strong internal validity and transparent predictions through SHAP-based explanations; however, the findings are not generalizable without independent clinical validation and prospective evaluation. Although this analysis used a stratified subset of 1500 records from a 100,000-entry publicly available dataset to preserve class distribution and demographic diversity, the cross-sectional design and synthetic origin limit its real-world generalizability. These constraints underscore the need for future research, including multi-center external validation, longitudinal modeling, calibration studies, and workflow integration assessments. Rather than claiming deployment readiness, this work provides a replicable template for responsible AI development in healthcare, emphasizing fairness, interpretability, and methodological rigor.

This study successfully addressed all stated objectives: (1) SMOTE effectively improved minority class detection, enhancing sensitivity to high-risk cases; (2) the Random Forest model achieved the highest predictive performance among the evaluated algorithms; (3) SHAP analysis provided clinically plausible global and local explanations that align with established medical knowledge; and (4) model stability and subgroup fairness were confirmed, supporting the potential for future clinical integration.

## Figures and Tables

**Table 1:** Comparative Analysis of Recent Diabetes Prediction Studies.

Study	Class Imbalance Handling	Interpretability Method	Dataset Size	AUC	Accuracy	Clinical Validation	EHR-Compatible Features	Real-Time Inference
Abnoosian et al. [6]	None	None	15,000	0.962	94.20%	X	X	X
Chowdhury et al. [4]	Ensemble SMOTE	None	8500	0.983	95.80%	X	X	X
Akshaya et al. [21]	None	LIME	~1200	0.950	~92%	X	X	X
Akhtar et al. [26]	SMOTE	XAI (LIME)	~1000	0.880	~88%	X	X	X
This Study	SMOTE + Leakage Prevention	SHAP (Global + Local)	1500	0.998	96.91%	✓ (Endocrinologist Review)	✓ (EHR-Extractable Features)	✓ (0.012 ms latency)

**Table 2:** Dataset Characteristics Summary.

Feature	Type	Description	Clinical Relevance	Missing (%)
Glucose	Continuous	Plasma glucose (mg/dL)	Primary diagnostic indicator	2.30%
BMI	Continuous	Body Mass Index (kg/m^2^)	Obesity risk factor	1.90%
Age	Continuous	Age in years	Demographic risk	0%
Blood Pressure	Continuous	Diastolic BP (mmHg)	Cardiovascular risk	4.70%
Insulin	Continuous	2 h serum insulin	Metabolic function	12.60%
Pregnancies	Discrete	Number of pregnancies	Gestational diabetes history	0%
Skin Thickness	Continuous	Triceps fold (mm)	Adiposity indicator	8.10%
Diabetes Pedigree	Continuous	Genetic predisposition score	Family history	0%

**Table 3:** Data Preprocessing and SMOTE Configuration.

Component	Parameter	Value	Rationale
Missing Data Imputation	<5% missing	Median imputation	Glucose, BP, BMI
	5–10% missing	MICE	Skin Thickness
	>10% missing	Multiple imputation	Insulin
SMOTE Configuration	*k*-neighbors	5	Optimized via grid search
	Sampling strategy	1:1 ratio	Balanced class distribution
	Random state	42	Reproducibility

**Table 4:** Performance Metrics.

Metric	Formula	Clinical Importance
Accuracy	(TP + TN)/(TP + TN + FP + FN)	Overall correctness
Precision	TP/(TP + FP)	Avoid false alarms
Recall	TP/(TP + FN)	Detect all diabetes cases
F1-Score	2 × (Prec × Rec)/(Prec + Rec)	Balanced performance
AUC-ROC	Area under ROC curve	Discrimination ability
Specificity	TN/(TN + FP)	Correctly identify healthy cases

**Table 5:** Clinical Performance Evaluation Framework for Diabetes Prediction Models.

Metric	Formula	Clinical Importance	Clinical Priority	Threshold for Clinical Acceptability	Literature Benchmark
Accuracy	(TP + TN)/(TP + TN + FP + FN)	Overall diagnostic correctness; important for general screening programs	Medium	≥85% for population screening	78–94% (diabetes prediction studies)
Precision (PPV)	TP/(TP + FP)	Minimizes false alarms; reduces unnecessary patient anxiety and healthcare resource waste	High	≥80% for clinical deployment	72–89% (clinical decision support)
Recall (Sensitivity)	TP/(TP + FN)	Detects all diabetes cases; critical for preventing missed diagnoses and complications	Very High	≥90% for disease detection	82–95% (medical screening tools)
Specificity	TN/(TN + FP)	Correctly identifies healthy individuals; prevents overdiagnosis and treatment burden	High	≥85% for population health	76–92% (preventive medicine)
F1-Score	2 × (Prec × Rec)/(Prec + Rec)	Balanced performance measure; optimal for imbalanced medical datasets	Medium	≥0.80 for clinical utility	0.75–0.88 (healthcare AI)
AUC-ROC	Area under ROC curve	Threshold-independent discrimination; guides clinical cutoff selection	High	≥0.85 for clinical implementation	0.82–0.94 (diabetes risk models)
NPV	TN/(TN + FN)	Confidence in negative results; crucial for ruling out disease in low-risk patients	High	≥95% for screening exclusion	88–98% (diagnostic tests)
Matthews Correlation	√[(TP × TN − FP × FN)/((TP + FP)(TP + FN)(TN + FP)(TN + FN))]	True correlation measure; unbiased performance assessment for imbalanced datasets	Medium	≥0.60 for reliable prediction	0.45–0.75 (medical classification)

**Table 6:** Performance Comparison of Machine Learning Models (Original vs. SMOTE-Enhanced).

Algorithm	Dataset	Accuracy (%)	AUC	F1-Score	Recall	Precision
Random Forest	Original	96.91	0.998	0.970	0.990	0.950
	SMOTE	96.67	0.997	0.970	0.960	0.970
Gradient Boosting	Original	95.70	0.993	0.950	0.960	0.940
	SMOTE	93.50	0.991	0.930	0.940	0.920
SVM	Original	83.00	0.840	0.800	0.830	0.780
	SMOTE	79.30	0.860	0.800	0.820	0.780

Note: All performance metrics are accompanied by 95% confidence intervals, available in the Supplementary Materials for statistical interpretation.

**Table 7:** Comparative Performance with State-of-the-Art Studies.

Study	Year	Method	Accuracy (%)	AUC	Key Innovation
This Study	2024	RF + SMOTE + SHAP	96.91	0.998	Balanced ensemble with explainability
Abnoosian et al. [6]	2024	XGBoost + Mahalanobis	94.20	0.962	Outlier removal
Kumar et al. [33]	2024	Ensemble SMOTE	95.80	0.983	Multi-classifier
Akhtar et al. [24]	2025	ML + XAI	~88.00	0.900	Transparent prediction
Akshaya et al. [27]	2025	Hybrid Ensemble	~92.00	0.950	Non-invasive EGG

**Table 8:** Diagnostic Performance Metrics for Random Forest.

Metric	Value	Clinical Interpretation
Sensitivity	99.50%	Excellent case detection
Specificity	97.30%	Minimal false alarms
PPV	96.20%	High confidence in positive prediction
NPV	99.70%	Strong rule-out capability
False Negative Rate	0.50%	Near-zero missed cases

**Table 9:** Cross-Validation Performance of Random Forest.

Fold	Accuracy (%)	AUC	Assessment
1	96.80	0.997	Excellent
2	97.10	0.999	Outstanding
3	96.50	0.998	Excellent
4	97.30	0.998	Outstanding
5	96.90	0.998	Excellent
Mean ± SD	96.90 ± 0.30	0.998 ± 0.001	Highly Stable

**Table 10:** Subgroup Performance of Random Forest.

Subgroup	Accuracy (%)	AUC	Sample Size
**Age**			
21–35 years	97.20	0.998	720
36–50 years	96.80	0.997	486
51+ years	96.40	0.996	294
**Gender**			
Female	97.10	0.998	980
Male	96.60	0.997	520
**BMI**			
Normal	95.80	0.994	312
Overweight	96.90	0.997	543
Obese	97.40	0.998	645

**Table 11:** Computational Performance of Machine Learning Models.

Algorithm	Training Time (s)	Prediction Time (ms	Clinical Suitability
Logistic Regression	0.12 ± 0.02	0.001	High-throughput
Decision Tree	0.18 ± 0.03	0.003	Rapid deployment
Random Forest	2.34 ± 0.21	0.012	Optimal balance
SVM	4.67 ± 0.45	0.025	Batch processing

## Data Availability

The dataset used in this study is available from the corresponding author upon reasonable request. The data are not publicly available due to institutional privacy policies.

## Supplementary Materials

The following supporting information can be downloaded at: https://www.mdpi.com/article/10.3390/healthcare13202588/s1, Table S1: Summary of Feature Elimination Process.
