# Supplementary material for: Interpretable Machine Learning Framework for Diabetes Prediction: Integrating SMOTE Balancing with SHAP Explainability for Clinical Decision Support
Source: Healthcare (Basel). 2025 Oct 14;13(20):2588. doi: 10.3390/healthcare13202588 (PMC12563896; doi:10.3390/healthcare13202588)
Supplement: Supplementary file 1 [file healthcare-13-02588-s001.zip › healthcare-3881962-supplementary.pdf]

healthcare

MDPI

Supplementary Materials

Table S1: Summary of Feature Elimination Process

Feature	Action	Reason
Insulin	Removed	Missing >12%, low SHAP value
Skin Thickness	Removed	High missingness, unstable
Diabetes Pedigree	Retained	High predictive value
